# Patient perspectives on the implementation of routinised syphilis screening with HIV viral load testing: Qualitative process evaluation of the Enhanced Syphilis Screening Among HIV-positive Men trial

**DOI:** 10.1186/s12913-021-06602-1

**Published:** 2021-06-30

**Authors:** Kinnon R MacKinnon, Ramandip Grewal, Darrell HS Tan, Rodney Rousseau, John Maxwell, Sharon Walmsley, Paul A MacPherson, Anita Rachlis, Nisha Andany, Sharmistha Mishra, Vanessa G Allen, Ann N. Burchell

**Affiliations:** 1 School of Social Work, York University, 4700 Keele Street, M3J 1P3 Toronto, Ontario Canada; 2 MAP Centre for Urban Health Solutions, Li Ka Shing Knowledge Institute, St. Michael’s Hospital, Unity Health Toronto, 30 Bond Street, M5B 1W8 Toronto, Ontario Canada; 3 Department of Medicine, University of Toronto, Toronto, Canada; 4 Toronto General Research Institute, University Health Network, Toronto, Canada; 5 Division of Infectious Diseases, St. Michael’s Hospital, Unity Health Toronto, Toronto, Canada; 6 Department of Immunology, University of Toronto, 1 King’s College Cir, M5S 1A8 Toronto, Ontario Canada; 7 AIDS Committee of Toronto, 543 Yonge Street, 4th floor, M4Y 1Y5 Toronto, Ontario Canada; 8 Toronto General Hospital, University Health Network, 200 Elizabeth Street, M5G 2C4 Toronto, Ontario Canada; 9 Division of Infectious Diseases, The Ottawa Hospital, 501 Smyth Road, L1H 8L6 Ottawa, Ontario Canada; 10 Ottawa Hospital Research Institute, Ottawa, Canada; 11 Department of Medicine, University of Ottawa, Ottawa, Canada; 12 Sunnybrook Health Sciences Centre, 2075 Bayview Avenue, M4N 3M5 Toronto, Ontario Canada; 13 Division of Infectious Diseases, Department of Medicine, University of Toronto, Toronto, Canada; 14 Institute of Medical Science, University of Toronto, Toronto, Canada; 15 Institute of Health Policy, Management, and Evaluation, University of Toronto, Toronto, Canada; 16 Public Health Ontario Laboratories, Public Health Ontario, 661 University Avenue, M5G 1M1 Toronto, Ontario Canada; 17 Department of Family and Community Medicine, St. Michael’s Hospital, Unity Health Toronto, 30 Bond Street, M5B 1W8 Toronto, Ontario Canada

**Keywords:** Sexually transmitted infections, HIV care, Syphilis, Men’s sexual health, Grounded theory, Qualitative interviews, Patient process evaluation

## Abstract

**Background:**

Syphilis infections have been on the rise, affecting men living with HIV in urban centres disproportionately. Since individuals in HIV care undergo routine blood testing, HIV clinics provide practical opportunities to conduct regular and frequent syphilis testing. Following the implementation of a routine syphilis testing intervention in HIV outpatient clinics, we conducted a qualitative process evaluation of patient experiences to measure patient acceptability, barriers to implementation, and facilitators of successful uptake.

**Methods:**

Upon completion of the trial, which took place at four HIV outpatient clinics in Toronto and Ottawa, Canada, we recruited male patients attending these clinics from November 2017 to April 2018. Interviews were conducted on-site and were audio-recorded and transcribed verbatim. All participants provided written informed consent. Interview data were analyzed using grounded theory, assessing qualitative modulators of effective uptake of routinised syphilis testing.

**Results:**

A total of 21 male patients were interviewed. Overall, interviewees found the clinical intervention acceptable, endorsing the practice of routinising syphilis testing alongside regular viral load bloodwork. Some men preferred, based on their self-assessment of syphilis risk, to opt out of testing; we considered this as a potential barrier to uptake of population-wide routinised syphilis testing. Interviewees also identified multiple facilitators of successful uptake, including the de-stigmatising of STI testing as a consequence of the universal nature of routinised testing. Participants recommended a routinised syphilis screening intervention to give patients peace of mind surrounding their sexual health. Participants identified HIV care clinics as comfortable and efficient locations to offer testing.

**Conclusions:**

Overall, most men were in support of implementing routinised syphilis testing as part of standard HIV care. From the patient perspective, HIV care clinics are convenient places to be tested for syphilis, and the routine approach was viewed to have a de-stigmatisng effect on syphilis testing.

**Trial registration:**

ClinicalTrials.gov NCT02019043; registered December 23, 2013.

**Supplementary Information:**

The online version contains supplementary material available at 10.1186/s12913-021-06602-1.

## Background

Rates of syphilis and other bacterial sexually transmitted infections (STIs) have increased dramatically among men living with HIV in North America, Europe, and Australia [1]. Frequent testing and early treatment can prevent adverse health outcomes associated with untreated syphilis and reduce the risk of transmission to partners [2]. International guidelines recommend syphilis screening for people with HIV at least once per year, with some advising more frequent testing at 3–6 month intervals [3–6]. *Canadian STI Guidelines* recommend screening for bacterial STIs, including syphilis, at 3-month intervals in the absence of symptoms for individuals identified as at ongoing risk [3]. Nevertheless, there are challenges to ensuring that those at ongoing risk have access to screening, particularly if testing decisions are based only on perceived risk by patients and clinicians, or signs and symptoms. Asymptomatic syphilis can occur, particularly among men living with HIV and among those who have previously had syphilis and are reinfected [7, 8].

In HIV care settings, there is a practical and efficient opportunity to conduct regular and frequent syphilis screening because blood tests are routinely ordered for HIV viral load monitoring. From 2015 to 2017, we conducted the Enhanced Syphilis Screening among HIV-positive Men (ESSAHM) Trial, which implemented an opt-out, clinic-based intervention to routinise syphilis testing with HIV viral loads. Specific objectives of the trial were to determine the degree to which the intervention: (1) increased the detection rate of early syphilis; (2) increased the proportion of men who undergo syphilis testing at least annually (screening coverage); and (3) increased screening frequency.

As part of the process evaluation plans of the trial, we sought to measure patient acceptability, barriers to implementation, and facilitators of successful intervention uptake, as well as to invite men to offer recommendations on improving syphilis screening frequency and coverage for men living with HIV. This was motivated by a desire to understand the results of the trial outcome evaluation, as well as to anticipate needs for sustaining and scaling-up routinised syphilis screening beyond the completion of the trial.

## Methods

The setting was four hospital-based HIV outpatient clinics in Toronto and Ottawa, the two largest cities in the province of Ontario, Canada. The ESSAHM Trial was a pragmatic, cluster-randomised controlled superiority trial using a stepped wedge design. Complete details are available in the published trial protocol [9]. Briefly, from February 2015 to July 2017, the intervention was gradually rolled out across clinics according to a randomised order; this was done such that, by the final six months of the trial, all clinics had implemented standing orders for syphilis testing with routine HIV viral loads. The trial study population included all men in HIV care regardless of recent sexual activity which may not always be known by clinicians; this pragmatic design feature eliminated the need for taking sexual histories [10]. Women in HIV care were not included in the intervention because, at the time that the trial was designed, there were no new cases of syphilis among women attending these HIV clinics [11] or in provincial surveillance data [12]. Prior to implementation of the practice change, syphilis testing practices were “usual care” as determined by physicians. For instance, tests were prompted by signs or symptoms, exposure to active cases, patient disclosure of sexual risk behaviour, and physicians’ experience-based knowledge of syphilis risk. Once clinics initiated the practice-change intervention, there were standing orders for syphilis serology whenever men underwent venipuncture for routine HIV viral load tests. Syphilis tests followed the practice of the centralized provincial laboratory in Ontario which conducts all syphilis testing using a reverse algorithm: (1) screen with chemiluminescent (CLIA) test; (2) if CLIA was reactive or indeterminate, additional testing was performed with the rapid plasma reagin (RPR) test, the Treponema pallidum Particle Agglutination (TPPA) test or the Fluorescent Treponemal Antibody Absorbance (FTA-Abs) test (9). Men diagnosed with syphilis would have received treatment and follow-up according to usual care. At any time during the trial, patients were able to opt-out of syphilis testing and/or refuse to have their clinical data used for the trial by so-advising their health care provider.

Following the completion of the ESSAHM Trial, between November 2017 and April 2018, we interviewed men living with HIV who were patients at each of the four trial sites during the intervention period. This ensured that interview participants could provide insights on the trial rooted in their personal experiences. All adult male patients were eligible for an interview, whether or not they had undergone syphilis testing or opted out from testing during the trial.

### Theoretical approach

For the qualitative process evaluation, we applied grounded theory methodology due to its rigorous approach to inductively generate theory about specific social phenomena. We followed the traditional, inductive grounded theory methodology originally developed by Glaser and Strauss to empirically study and give meaning to a given social issue, contextualising how social phenomena interact with the wider environment [13]. Of note, although some contemporary grounded theories may involve hypothesis testing, Glaser and Strauss (1967) discouraged imposing prior concepts on data, and they also “warned against logico-deductive methods that begin with hypotheses that researchers cannot change but either accept or reject them when the analysis is concluded” [14]. By iteratively and dynamically collecting and analysing more context-specific data related to the research question, data serve to construct knowledge about the topic being studied [15, 16].This approach aligns with our aim of iteratively developing a holistic understanding, grounded in the experiences of men living with HIV, and of the human factors that may limit or enhance successful implementation of routinised syphilis testing.

### Researcher reflexivity

Practices of reflexivity implore researchers to establish transparency throughout the research process, and to identify their social, cultural, and political positionality in relation to the content area under study [17]. This is important because researchers’ positionalities as either an insider or outsider to the population under study may affect data collection and analysis. In addition to collaboratively developing the interview guide with the ESSAHM Trial Steering Committee, first author and interviewer (KMK) engaged in reflexive exercises—such as critical self-reflection—throughout the data collection, analysis, and writing stages [18]. Specific to qualitative interviewing, when interviewers take the position of an insider, this may contribute to the perception of significant shared understanding of the topic between interviewer and participant and thus important details going unspoken, yielding worse quality data [19]. Given the study population, discussing the interviewer’s HIV status or syphilis testing experiences could affect participant interview responses, as this practice contributes to the interviewer being positioned as either insider or outsider [20]. To enhance rigour in data collection, KMK did not position himself as HIV positive or negative, and did not discuss any syphilis testing experiences with participants during the recruitment or data collection stages. Furthermore, prior to conducting interviews KMK was not involved with the ESSAHM Trial and did not have any previous contact with the HIV clinics included in the trial, or with any of the trial patients or interview participants.

### Recruitment and data collection

We recruited patients using methods consistent with grounded theory [21]. To mitigate the risk of potential site-specific bias, we recruited from all four study sites a diverse sample of men (n = 21). A designated staff member at each clinic approached male patients to inform them about the process evaluation study, inviting them to volunteer to be interviewed. Interested men were directed to a private interview room where KMK explained the study in greater detail. The interviewer described the procedures used for the ESSAHM Trial procedures and provided the original trial information pamphlet to review (Supplemental Material) which included general facts about syphilis, including that it was on the rise among HIV-positive men in Ontario, that untreated syphilis may have harmful impacts such as neurosyphilis, that it can be detected with a simple blood test, and that it can be treated. All participants provided written and informed consent to participate.

Interviews of variable length (8 to 36 min) were conducted with 21 patients across the four sites. Interview content was audio-recorded and transcribed verbatim. Interviews were semi-structured and followed an interview guide that was collaboratively created by two of the ESSAHM co-PIs (ANB, DHST), KMK, and RG, and in consultation with members of the ESSAHM Trial Steering Committee (VA, SG, JM, SM, PAM, AR, RR and SW). The guide invited men to share their experiences and opinions of routine syphilis testing and their perceptions on the potential best practices of future clinic-based syphilis testing (Supplemental Material). Sample interview questions included: “What are your thoughts on having your blood routinely tested for syphilis as part of your regular blood work, as opposed to doing it only when you or your health care provider thinks it is necessary?”; “Which benefits, if any, did you experience as a result of this routine testing program?”; “If you were to discuss regular syphilis testing blood work with your friends, what would be the benefits or drawbacks you would mention?”; “How should patients go about opting in or opting out of routine testing?”; and “Going forward what do you think about regular syphilis testing being offered as part of the routine/regular practice here at (name of clinic)?”. These questions also included several related probes to facilitate an open and complex conversation.

### Data analysis

Several strategies were employed to validate the trustworthiness of relationships between raw data and findings [22, 23]. Data were analysed iteratively through several phases of interview transcript coding and theoretical memo-writing completed by KMK and the study coordinator, RG. Memo-writing facilitated a deeper understanding of how participants felt about routinised syphilis testing, with the topic of syphilis testing, and how the prevalent themes in the data compared to one another. These theoretical memos also contributed to the development of the coding framework. We inductively analysed interview transcripts using grounded theory’s constant comparative method, through which analysis occurs in three main stages: (1) open coding; (2) selective coding and theoretical memo-writing; and (3) theoretical sampling to reach saturation. The constant comparative method enabled the identification of common themes across interview transcripts, the creation of a coding framework, and finally, confirming the repetition of themes across interviews which signals theoretical saturation [18, 21].

The qualitative data analysis software *NVivo* was used to assist with dataset management, to iteratively generate the coding framework, and to code and analyse the data. Prior to using *NVivo*, KMK and RG independently reviewed and open coded transcripts from the first 18 interviews at three of the four clinic sites, and then collectively discussed the primary themes emerging from the dataset. Data were then categorised and described. In comparing open coding notes, the major emergent themes were highlighted to construct a coding framework that could be used to selectively code the data. The emerging themes and coding framework were presented to study PI, ANB, who also reviewed all interview transcripts. The coding framework was then used to conduct selective coding using *NVivo* analysis software prior to conducting the final three interviews at the fourth trial intervention site. Because the final three participants discussed experiences and ideas consistent with previous interviews at the other sites, we concluded that theoretical saturation had been reached and data collection was complete. Although interview transcripts were labeled according to the site at which they were obtained, each transcript was subsequently reviewed independently to further assess how the men uniquely understood and engaged with the topic of routine syphilis testing. No major differences in the men’s experiences or ideas were revealed between the trial clinic sites.

## Results

### Barriers to Implementing routinised syphilis testing

### Opting out based on individual perception of risk

 Although nearly every participant interviewed endorsed routine syphilis testing as standard practice at HIV clinics, some men qualified this recommendation with reservations. A few men, who were either sexually abstinent or in long-term monogamous partnerships, noted that they did not think their unique situations warranted routine syphilis testing, and for that reason they should be able to opt out. One man shared, for example: “I’ve always been negative of syphilis; I don’t know, I think, my degree of risk isn’t consistent, intermittent, so…I think the test should reflect that. Just in my case, anyway.” (Participant 18, Clinic 3). Another man, who identified as straight, mentioned his awareness of syphilis being more prominent among men who have sex with men, and that therefore he did not feel that routine testing was necessary for him. Overall, these men still associated a plethora of benefits with routinising syphilis testing, acknowledging that it is in the public’s best interest. Nevertheless, some men qualified that certain personal circumstances warranted the option to opt-out when risk is low or intermittent.

Men provided a range of explanations as to why they might not fit the risk profile for syphilis infection. One man, as an outlier example, reported that engaging in condomless anal sex would alone not prompt him to seek syphilis testing, but that he imposes his own self-determined set of criteria surrounding sexual activities, risk, and STI transmission. On the other hand, some men who identified as being in committed monogamous relationships, and who reported that their partners were not engaging in sexual activity outside of the relationship, expressed personal reluctance to test and as such endorsed the opt-out approach. Consequently, the routinising of syphilis testing may face implementation barriers due to participants opting out by self-assessment of risk.

### Facilitators of Successful Uptake

#### Offering syphilis testing as part of HIV care provides comfort and efficiency

Most participants reported past experiences of STI testing at numerous and varied sites including local sexual health clinics, community centres, bathhouses, and primary care providers’ offices. Men were directly asked where they would prefer to be tested for syphilis. Most men expressed comfort attending a sexual health clinic, but, for several reasons, preferred testing at their regular HIV care clinic; participants reported feeling more comfortable there and more trusting of the staff to be knowledgeable and “non-judgmental”. A number of men pointed out that they were already having regular blood work at the HIV clinic, so having an extra vial of blood taken for testing was a more efficient way of having syphilis testing done. This testing approach was reported to reduce the stress of remembering when one last had a syphilis test. To this end, a syphilis test at each regular clinic visit may lift the burden of responsibility to remember syphilis testing. One man shared that:

I find, again, I really liked it here [HIV care clinic] because it’s somewhere I go regularly. And I get blood work done on a regular basis. And I’ve built a relationship with the people that work here so I feel comfortable with them. (Participant 16, Clinic 3).

#### Men’s awareness of the syphilis epidemic

A great proportion of men interviewed expressed knowledge of the growing number of syphilis cases in Canada, and related this to the importance of syphilis testing.

Syphilis is rising so much here in Canada, so it would be a great opportunity, to give people chance to go through the routine [test] every time they do their blood culture. You know, just to monitor… if just, the result come positive, you know, how to prevent not to… not to spread it further. (Participant 1, Clinic 2)

Some interviewees attributed the phenomenon of rising syphilis infections with condomless sex practices. Of note, this connection was articulated primarily by those who openly discussed within interviews their sexual identities and connections to gay, bisexual, and other men who have sex with men (gbMSM) communities. These men, in particular, identified the increasing rates of syphilis infections and noted seeing public service announcement posters in their HIV care clinics and other sexual health clinics. Some men expressed concerns about a perceived rise in frequency of condomless sexual activities attributable to the availability of pre-exposure prophylaxis (PrEP). For instance, one man extrapolated that:

Well, my HIV community, the subculture, most people that have HIV are exposing their self, to other things. Syphilis, chlamydia, and, it sort of goes together, right? … There’s this, I’m safe now attitude, right? So people are less likely to take protection where others might. So you’re exposing yourself on a regular basis, so, routine [syphilis testing], it should just be routine, when you’re tested, right? (Participant 2, Clinic 1)

Another man similarly noted:

Since I guess, Truvada came out with the PrEP, and all that stuff. It [syphilis] seems to be spreading like rapid fire… it’s given them [men] confidence that they’re not gonna contract HIV. So then it opens the door to so much more [STIs]. (Participant 17, Clinic 3)

Thus, the growing number of syphilis cases was noted as a major justification for routinising syphilis testing.

#### Reducing stigma associated with STI testing

Despite that fact that all participants were receiving ongoing HIV care, many continue to confront difficulties in requesting syphilis testing due to pervasive stigma [24, 25]. Specifically, when asked if he felt he could request a syphilis test, one man replied:

Not at my doctor’s. I would have, at the sexual health clinic, which would have been a much more, that’s an anonymous type of place… In a certain way, it was always a little risky to go to the sexual health clinic because it still requires going into a building… (Participant 4, Clinic 2).

As a result of these patient-side barriers to initiating STI testing, patients viewed routinising syphilis testing as a mechanism to reduce stigma. In this way, reducing stigma and fears related to accessing testing operates as a facilitator to routine syphilis testing. For example:

It [routinised syphilis testing] makes it easier for me, I don’t feel embarrassed about asking for a specific test, but if I was, having my healthcare provider bring up the option of getting tested for that, alleviates any sort of discomfort when having to discuss it. Because it’s just being done with everyone. It’s just routine, right? (Participant 2, Clinic 2).

#### Ensuring that syphilis testing is not forgotten

The way that HIV commands both patient and providers’ attentions in HIV care encounters, to the detriment of other sexual health issues, was another theme that emerged as a potential barrier to syphilis testing that routinisation would overcome. One man explained that, while he was receiving care for HIV (presumably before the clinic had implemented the practice of routinised syphilis testing), he had not been tested for syphilis, delaying his eventual diagnosis:

As it turned out, we discovered that I had fallen through the cracks. I was not being tested regularly for syphilis either here or with my GP. He just assumed that at, through the clinic…It was happening, right? And it wasn’t, right? So we had no idea how long I’d been infected [with syphilis]. (Participant 12, Clinic 3)

The implementation of routinised syphilis test therefore overcomes the potential for missed testing caused by the assumption that “someone else is taking care of it” or the risk of falling “through the cracks”.

#### Offering peace of mind

A majority of men interviewed associated routine syphilis testing with comfort surrounding their health status. In fact, several men referred to routine syphilis testing as offering “peace of mind”. One interviewee stated “You get peace of mind and if… if they find something you can get at it quicker.” (Participant 5, Clinic 2). Similarly, when asked about the benefits of routine syphilis testing, another interviewee reported that “the key benefit is being tested and knowing whether you have [syphilis] or not… If you have it, then you can get treated and if not, then, you know you’re good for a while” (Participant 4, Clinic 2). Acknowledging that early identification of infection may help reduce the frequency of onward transmission of syphilis, one man noted that routinised testing may improve overall sexual health in the greater population, even if he did not perceive an explicit individual benefit of the ESSAHM Trial:

I can see the benefit, had I contracted it and treated it, I’d like to know so that I can take care of it and clear it up… But no, I mean I haven’t, specifically myself, had a benefit but I’m sure, other people being tested, if they did contract it and then treating it, it probably benefits me in the long run, right? (Participant 14, Clinic 3).

Another man, who was diagnosed and treated for syphilis during the ESSAHM Trial, stated that:

It was treated, it was done quickly. And it was done without anybody having to guess that there was something wrong. So it was nice that it was just part of the routine and saying, OK, like you’re getting your kidneys checked… (Participant 1, Clinic 1).

In other words, many participants expressed that they could take comfort, and feel reassured, knowing that they were being tested regularly for syphilis, rather than being tested intermittently based solely on risk perception by themselves or by a care provider.

### Patient recommendations

Participants were invited to provide recommendations related to syphilis testing, both within the context of routinised testing as evaluated by the ESSAHM Trial, and for better syphilis testing services more broadly. Men explained that they depend on their care providers to inform them about the latest health concerns, such as the importance of syphilis testing. This included a desire for providers to encourage patients to engage in regular syphilis testing, and to inform them of the potential disadvantages of not being tested. One man, for example, indicated his deference to clinician judgment surrounding testing, and reliance on his care providers’ clinical knowledge:

Because you, as a patient, are completely ignorant of medical practice, you have to rely on the person on the other side of the table, that they’ve got the knowledge… So should it be done? Completely up to the doctors, I think. (Participant 2, Clinic 4).

Some interviewees further recommended that syphilis testing be promoted using public service announcements/posters in community event-related spaces in which large groups of men gather (e.g. bathrooms at sporting events). Men noted the importance of communicating information about syphilis testing broadly, using multiple methods.

Relatedly, some men perceived that testing for HIV had, in the broader community, taken priority over testing for other STIs. In effect, HIV testing has become widely available for HIV-negative men, but testing for other STIs is comparatively more difficult to access. A participant shared, for example, that HIV testing is readily available in some community centres and other relevant sites such as a local campground that caters to gbMSM patrons. These services were not understood to offer bacterial STI testing alongside HIV testing, making this sexual health service non-inclusive or relevant to men living with HIV. It was therefore recommended to offer syphilis and other STI testing distinctly, but alongside such HIV testing services: “I don’t think they do syphilis testing…I think it’s just HIV testing. So the encouragement would be to have, when you’re gonna do testing, do all the round, testing, test it all” (Participant 4, Clinic 2). Thus, participants recognised that other sexual health issues tend to come secondary to HIV testing, and that STI testing such as for syphilis should be prioritised.

## Discussion

Our grounded theory process evalution of a routisined syphilis screening intervention in four HIV clinics in Ontario, Canada, revealed patient opinions that are important to consider for implementation, long-term sustainability, and scale-up of such interventions. Overall, the patients interviewed supported routine syphilis testing as part of standard HIV care. This high acceptability is in keeping with reviews of the effectiveness of STI testing interventions, where trials of routine testing approaches predominate; these are typically found to be effective as defined as significant increases in the proportion tested or the frequency of testing [26, 27]. Indeed, in the ESSAHM Trial, we observed a three-fold increase in the odds of having at a least one syphilis test per year, and a doubling in the number of tests per year [28]. Our application of grounded theory, together with critically reflecting on our positionality and insider/outsider status in relation to the patient population and research process, offers novel study contributions to the implementation science literature. Given that interviewer, KMK, did not explicitly position himself as an “insider” with the participants in terms of HIV positive status, men interviewed may have presumed him to be an “outsider” to the community. Such a dynamic strengthens the trustworthiness of our findings in that participants felt comfortable strongly endorsing routine syphilis testing with someone who may have been perceived as being outside of their own lived experiences.

In terms of facilitators, the men we interviewed recognised the impact of the syphilis epidemic on health and community wellness. Among participants who disclosed sex with other men, in particular, we noted an awareness of rising rates of syphilis diagnosis. This perception was accurate according to syphilis case reporting, which in 2017 occurred at rates of 67.8 per 100,000 males in Toronto and 19.7 per 100,000 males in Ottawa [29]. Moreover, in a large cohort in Ontario involving the participating HIV clinics, new syphilis diagnoses occur at minimum rates of 4 syphilis cases per 100 person-years [11, 30]. Interviewees described this phenomenon as attributable to a rise in condomless sex. Routine syphilis testing, as suggested by many of the men interviewed, is an important mechanism to improve men’s access to testing.

In comparison with risk-based targeted testing approaches, routine testing may be one potential mechanism for reducing stigma surrounding syphilis and other STIs. The potential for de-stigmatisation was a motivator for the ESSAHM Trial, because testing all patients minimises the perception that any one individual is being singled out [9]. Nevertheless, our team was concerned that men might perceive that the approach would perpetuate stigma because it targeted testing to men living with HIV. So it is worth noting that men we interviewed endorsed the idea that routinising syphilis testing could in fact have a de-stigmatising effect because “it’s just being done with everyone. It’s just routine…”. While previous studies found that some men are hesitant around STI testing, and associate testing with shame and fear encountering homophobia [2, 31, 32], in our study men did not share these same fears. For this reason, we argue that routinising syphilis testing may circumvent the stigma experienced when patients ask for testing, or when providers recommend testing based on a patient’s ‘risk’ profile.

Ensuring that routine testing is paired with a non-judgmental approach to care is important for acceptability and scalability of this intervention. The men we interviewed noted that their HIV clinics and care providers produce a comforting atmosphere surrounding testing. Patient concerns regarding stigma, and healthcare provider fears of being perceived as judgemental or feeling uncomfortable discussing sexual practices, are documented barriers to STI care [2]. Approaches that de-stigmatise STI testing are well understood, and include sensitivity training for clinicians and structural changes that normalise STI testing by, for example, displaying STI educational materials and offering testing in clinic settings beyond sexual health clinics, such as in HIV care settings and primary care [33].

Men viewed HIV care clinics as the most efficient place to be tested for syphilis because they already have regular blood work done there. The ease through which syphilis testing could be integrated translated into “quicker” treatment, when necessary. Previously-identified patient barriers to STI testing include inconvenience, forgetting, and desire for anonymity or privacy. Alternatively, provider barriers include lack of time and competing priorities [2]. The results of our patient process evaluation extend this literature, indicating that syphilis testing performed in connection with regular HIV blood work may mitigate these barriers.

Our study also revealed potential pitfalls for the implementation of opt-out routinised syphilis testing paired with HIV viral loads. This approach seeks to overcome under-testing that may occur if men undergo testing based solely on provider- or self-assessment of risk (which may or may not be accurate [34]) or based on symptoms (which may not be present [7, 8]). Yet for ethical reasons, procedures that recognise men’s autonomy should be incorporated in the implementation of this intervention. Although the ESSAHM Trial team sought to maximise uptake, it also provided for a patient’s right to opt out from syphilis testing, as one may do for any medical procedure. Indeed, during process evaluation interviews, some men preferred to opt out of testing because they perceived their risk to be low. In the ESSAHM Trial, among 3,937 men attending the four participating clinics, only 1 % of men (n = 42) asked that their data not be included in the study. However, 26 % of submitted viral loads still did not have a corresponding syphilis test when all clinics had implemented routinised testing [28]. Because reasons for non-compliance were unmeasured, when men were not tested, we do not know whether this was because men did not receive a copy of the laboratory test requisition for syphilis or because men opted not to have the test done.

There are limitations worth noting. First, as with all qualitative grounded theory studies, there may be issues generalising our findings in other populations and in other contexts —particularly in non-urban settings and in different healthcare systems. That the vast majority of men we interviewed were in favour of syphilis testing paired with regular HIV blood work suggests that this could be an acceptable intervention to scale-up in clinical settings beyond the ones included in our trial, such as in primary care where HIV care is increasingly delivered [34]. Men were not asked to disclose their sexual orientation during interviews. Based on self-disclosure, most participants were gbMSM, with only two men disclosing that they were heterosexual. None of the interview volunteers had refused to be part of the trial. Had we been able to recruit and interview men who had refused, or more heterosexual men, we may have gathered different information on barriers, facilitators, and recommendations surrounding syphilis testing in HIV care clinics.

## Conclusions

Routinising syphilis testing with HIV viral loads, as we evaluated in the ESSAHM Trial, is an intervention that was acceptable to men living with HIV, allowing testing to be improved by de-stigmatising syphilis detection protocols and enhancing the likelihood of diagnosing cases earlier amongst HIV-positive men. Men were in support of implementing syphilis testing as part of standard HIV care, yet emphasised that the option to decline syphilis testing was important to maintain. To minimise opt-outs, we recommend that clinics preparing to implement this intervention pay due attention to the recommendations men offered here, including: having accessible health promotion materials that explain the importance of syphilis testing; ensuring that testing occurs in welcoming, compassionate, and non-judgemental environments; and emphasising the convenience of testing when one is already having bloodwork done.

## Supplementary Information

**Additional file 1:**

## Data Availability

The datasets (which includes individual transcripts) generated and analysed during the current study are not publicly available due to confidentiality policies given the sensitivity of patients’ personal health data surrounding sexual health, sexually transmitted infections, and HIV status. De-identified data may be made available by the corresponding author on reasonable request.

## Abbreviations

ESSAHM Trial: Enhanced Syphilis Screening Among HIV-positive Men Trial
gbMSM: Gay, bisexual, and other men who have sex with men
PI: Study principal investigator
PrEP: Pre-exposure prophylaxis medication
STI: Sexually transmitted infections
