# Supplementary material for: Patient perspectives on the implementation of routinised syphilis screening with HIV viral load testing: Qualitative process evaluation of the Enhanced Syphilis Screening Among HIV-positive Men trial
Source: BMC Health Serv Res. 2021 Jun 30;21:625. doi: 10.1186/s12913-021-06602-1 (PMC8243864; doi:10.1186/s12913-021-06602-1)
Supplement: Supplementary file 1 — Additional file 1: [file 12913_2021_6602_MOESM1_ESM.docx]

**Interview Guide – ESSAHM Clinic Site Patient Participants**

**Preamble: Prior to each interview participants will have an extensive discussion of the ESSAHM intervention project through a verbal exchange, reviewing information pamphlets, and by reading the informed consent form prior to agreeing to volunteer for the study. Each participant will be given the opportunity to ask questions about the ESSAHM study prior to agreeing to participate in an interview.**

1. I’m wondering how long you have been a patient of _______ (clinic site).
2. Before today, were you aware of the change in clinic practice to order syphilis tests with viral loads?

Probe: How did you find out?

Probe: Did your health care provider/health team discuss syphilis screening with you?

Probe: What did you understand from this conversation?

1. Were you aware that a research study was evaluating how well pairing syphilis testing along with HIV viral load tests works to detect new cases of syphilis?

Probe: Why was this study being done?

Probe: Did your health care provider discuss reasons for participating in the study?

Probe: What did you understand from this conversation?

Probe: Did you understand the difference between opting out of the testing versus opting out of having your data used to evaluate if regular testing works?

1. IF participant was aware:

Why *did/didn’t* you agree to have this additional blood test done?

IF participant was NOT aware: If you had known would you have opted out?

1. How should health care providers/health teams get permission from patients to do routine syphilis testing? E.g. having it done at the same time as other regular blood tests

Probe: How should patients go about opting in or opting out of routine testing?

Probe: What do you think about asking for written versus verbal permission for routine testing?

Probe: Do you think asking for permission should be different in a research study versus in regular clinical care with your health care provider?

1. Did the tests ever show that you had a new syphilis infection? (If no, skip probes… If yes…)

Probe: When did you learn you had a new syphilis infection? OR

Probe: Over the ____(past 6 months, 12 months, etc)____did your test show that you had a new syphilis infection?

Probe: Were you happy with the way that you found out?

Probe: Would you prefer that the results were communicated to you in another way? --If yes, please tell me how.

1. Had you ever been tested for syphilis before the clinic changed their practice ___________ (date)?
2. What are your thoughts on having your blood routinely tested for syphilis as part of your regular blood work, as opposed to doing it only when you or your health care provider thinks it is necessary?
3. What do you think are health care providers’/health team roles in syphilis testing?
4. I’m wondering which benefits, if any, you experienced as a result of this from routine testing program?

Probe: How about any negative outcomes as a result of this routine testing program?

1. Going forward what do you think about regular syphilis testing being offered as part of the routine/regular practice here at (name of clinic)? Is this a practice that should be continued?

Probe: Why or Why not?

1. Where else would you want to have syphilis testing done?

Probe: At ____(name of clinic)?

Probe: With your family doctor?

Probe: At a sexual health clinic (like Hassle Free)?

1. If you were to discuss regular syphilis testing blood work with your friends, what would be the benefits or drawbacks you would mention?
2. How can syphilis and other STI testing with men be improved?

Probe: What do you think would be the best way for men to get regular syphilis and other STI screening?
